# Critical Care After Thrombolytic Therapy in Acute Stroke: Who Really Needs the ICU?

**DOI:** 10.3390/jcm15010324

**Published:** 2026-01-01

**Authors:** Katherine G. Moore, Nathaniel S. Harshaw, Samantha K. LaRosa, Daria Indeck, Danielle Cross, Nicole Chiota-McCollum, Lindsey L. Perea

**Affiliations:** 1 Department of Surgery, Division of Trauma and Acute Care Surgery, Penn Medicine Lancaster General Health, 555 N Duke Street, Lancaster, PA 17602, USA; katemoore130@gmail.com (K.G.M.); nate.harshaw@gmail.com (N.S.H.); samanthalarosa88@gmail.com (S.K.L.); daria.indeck@pennmedicine.upenn.edu (D.I.); 2 Department of Neurology, Penn Medicine Lancaster General Health, 555 N Duke Street, Lancaster, PA 17602, USA; danielle.cross@pennmedicine.upenn.edu (D.C.); nicole.chiota-mccollum@pennmedicine.upenn.edu (N.C.-M.)

**Keywords:** stroke, ICU care, thrombolysis, tPA, Tenecteplase, ischemic stroke

## Abstract

**Background/Objectives**: Intravenous thrombolytic therapy remains the cornerstone of managing acute ischemic stroke (AIS) patients. Given the potential adverse effects of thrombolysis, patients are admitted to an intensive care unit (ICU) for close monitoring following administration. Alternative post-thrombolytic pathways may provide safe, cost-effective care in certain populations. We aimed to determine the proportion of patients treated with thrombolytics who required ICU care for reasons other than frequent neurologic monitoring and to define their characteristics. **Methods**: We retrospectively (May 2020–August 2022) reviewed patients ≥ 18 years of age who received Tenecteplase (TNK) or tissue plasminogen activator (tPA) for AIS at our stroke center. Patients were classified as requiring ICU care if they required intubation within 24 h of admission, required neurosurgical intervention, had symptomatic hemorrhagic conversion or brain compression, required a continuous infusion for hemodynamic management, or were in status epilepticus. Univariate and multivariable statistical analyses were performed. The study protocol was deemed exempt by our Institutional Review Board. **Results**: 262 patients met inclusion criteria. A total of 54 (20.6%) required ICU care. Multivariable analysis showed that patients on antithrombotic therapies prior to arrival (AOR: 3.344, *p* = 0.002) or who presented with higher initial NIH stroke scale (AOR: 1.116, *p* < 0.001) had a significantly higher likelihood of requiring an ICU level of care. **Conclusions**: In our cohort, approximately 21% of patients required critical care. Antithrombotic therapy before admission and greater NIH stroke scale on arrival were associated with an increased likelihood of requiring ICU care. Further prospective studies are indicated to assess the efficacy of alternative settings for post-thrombolytic care in selected AIS patients; however, our findings suggest that a specific subset of patients with AIS can be safely and effectively cared for in a non-ICU setting. This may have implications for the provision of safe, effective care while optimizing healthcare resource utilization.

## 1. Introduction

Thrombolytic therapy involves the use of Tenecteplase (TNK) or tissue plasminogen activator (tPA) to break up or dissolve blood clots. It remains the cornerstone of acute ischemic stroke (AIS) management for eligible patients and is associated with better functional outcomes when administered promptly [1,2,3]. Currently, tNK/tPA is administered within a 3–4.5 h window following symptom onset; however, there is still debate regarding the efficacy of administering tNK/tPA in an extended window, as administration further from symptom onset has been associated with a greater risk of developing symptomatic intracerebral hemorrhage (sICH) and mortality [4]. In current practice, once patients are administered this medication, they are typically admitted to the intensive care unit (ICU) for close monitoring of potential side effects, including sICH, angioedema, systemic hemorrhage, and the risk of subsequent hemodynamic instability [1,2]. As a result, our institution, as well as many others, routinely monitor post-thrombolysis patients in the ICU for at least 24 h, regardless of their initial clinical stability.

A recent study suggests that the risk of sICH is greatest in the 12 h following TNK/tPA administration and is negligible after this period [3]. Therefore, this study, among others, questioned whether prolonged ICU-level care is necessary for all post-thrombolysis patients, particularly those without early neurologic deterioration or need for invasive interventions.

The aim of this retrospective study was to elucidate the proportion and characteristics of patients who required critical care management and ICU procedures during the 24 h post-thrombolytic therapy among our community-based patient population, compared to those who required only close neurological and hemodynamic monitoring without ICU-specific interventions. We hypothesized that a sizeable proportion of post-thrombolysis patients do not require critical care and therefore could be safely cared for in a setting such as a progressive care unit with low nursing ratios and close clinical monitoring alone.

## 2. Materials and Methods

We conducted a retrospective review of all patients receiving intravenous (IV) thrombolysis for acute ischemic stroke at our thrombectomy-capable community-based stroke center between 1 May 2020 and 1 August 2022. The study protocol was approved by our Institutional Review Board (IRB) and, due to the retrospective nature of the study, we obtained a waiver of consent from the IRB. All patients ≥18 years of age who received Tenecteplase or tPA and had a final discharge diagnosis of acute ischemic stroke were included in the study. Patients were excluded from this study if they were under the age of 18 and if they did not have a hemorrhagic stroke diagnosis. Patients were retrospectively identified from our institution’s stroke database. Additional data came from our hospital’s electronic medical record. All data were collected and stored in REDCap version 15.8.3 (Nashville, TN, USA).

Statistical tests were conducted based on dichotomizing the study population into categories of those requiring an ICU level of care and those not requiring an ICU level of care. Patients were defined as requiring an ICU level of care if they required intubation within 24 h of admission, required neurosurgical intervention, had symptomatic hemorrhagic conversion or brain compression, required a continuous infusion for hemodynamic management, or were in status epilepticus. Patients were defined as not requiring an ICU level of care if they did not meet any of the aforementioned criteria. We then conducted analyses comparing a variety of demographic and patient outcome variables between groups. The variables of interest compared between groups included age, sex, race, in-hospital mortality, stroke etiology (small vessel, cardioembolic, larger artery, other identified source, unclear etiology), antithrombotic use, stroke on presentation (versus during hospital admission), mechanical thrombectomy, time from symptom onset to thrombolytic administration, systolic blood pressure at time of thrombolytic administration, National Institutes of Health Stroke Scale (NIHSS) at time of presentation and following TNK/tPA administration, hospital length of stay, ICU length of stay, discharge destination (home, skilled nursing facility, rehabilitation center, hospice), and 30-day readmission for stroke-related symptoms.

We used the statistical software Stata/IC 16.0 (College Station, TX, USA) for statistical analysis. For univariate analysis, we performed independent *t*-tests for continuous variables and chi-squared tests for the categorical variables to compare the characteristics and outcomes between those requiring an ICU level of care and those not requiring an ICU level of care. The results of the independent *t*-tests were reported as median (interquartile range [IQR]), *p*-value and the results of the chi-squared tests were reported as n (percent of population), *p*-value. We also performed a multivariable logistical regression, assessing the risk of requiring an ICU level of care while controlling for a variety of factors such as age, race, sex, stroke etiology, antithrombotic use, and initial NIHSS. Data for the multivariable logistic regression were reported as an Adjusted Odds Ratio [95% Confidence Interval], *p*-value. A *p*-value < 0.05 was considered significant.

## 3. Results

A total of 262 patients met the inclusion criteria. Based on our definition, 54 (20.6%) patients required an ICU level of care. Characteristics of those requiring an ICU level of care versus those not requiring an ICU level of care are described in Table 1. Of note, there was no significant difference between groups in age (no ICU: 71.5 (63–82), ICU: 74.5 (67–83), *p* = 0.4306) or proportion of male sex (no ICU: 26 (48.15%), ICU: 76 (36.53%), *p* = 0.721). Interestingly, a greater percentage of patients who did not require ICU care had a stroke on presentation to the hospital (rather than during hospitalization) compared to those requiring ICU-level care (no ICU: 204 (98.08%), ICU: 47 (87.04%), *p* < 0.001). A greater percentage of patients who did not require the ICU had a small vessel stroke compared to those requiring an ICU level of care (no ICU: 33 (15.87%), ICU: 1 (1.85%), *p* = 0.006). Conversely, a greater percentage of patients requiring ICU-level care had a large artery stroke compared to patients not requiring ICU care (no ICU: 86 (41.35%), ICU: 35 (64.81%), *p* = 0.002). There was no association between ICU requirements and cardioembolic stroke (*p* = 0.107), strokes with another identified source (*p* = 0.469), and strokes with an unclear etiology (*p* = 0.068). Of the 95 patients who received antithrombotic therapy before admission, a greater number did not require ICU-level care (no ICU: 64, ICU: 31, *p* < 0.001); however, 30.77% of the overall patients not requiring the ICU (n = 208) had pre-admission antithrombotic therapy compared to 57.41% of overall patients requiring an ICU level of care (n = 54) receiving pre-admission antithrombotic therapy.

A significantly greater proportion of patients defined as requiring an ICU level of care also underwent mechanical thrombectomy, as compared to those who were defined as not requiring an ICU level of care (no ICU: 16 (7.69%), ICU: 23 (42.59%), *p* < 0.001). Additionally, the group that required an ICU level of care had a significantly greater NIHSS initially (no ICU: median 6 (IQR 3–11), ICU: median 13 (IQR 7–18), *p* < 0.001) and a significantly greater NIHSS following TNK/tPA administration (no ICU: median 3 (IQR 1–7), ICU: median 13.5 (IQR 6–20), *p* < 0.001). Notably, there was a significant association between requiring ICU-level care and discharge destination (*p* < 0.001); a greater percentage of patients requiring an ICU level of care were discharged to skilled nursing facilities (no ICU: 26 (12.50%), ICU: 8 (14.81%) and hospices (no ICU: 3 (1.44%), ICU: 9 (16.67%) compared to those not requiring the ICU.

The results of the multivariable analysis assessing risk of requiring an ICU level of care are shown in Table 2. This analysis suggested that patients on antithrombotic therapies prior to arrival had a significantly higher likelihood of requiring an ICU level of care compared to those not on these therapies (AOR: 3.344; [95% CI: 1.544–7.242], *p* = 0.002). Patients with a higher initial NIHSS also had a significantly higher likelihood of requiring an ICU level of care (AOR: 1.116; [95% CI: 1.062–1.173], *p* < 0.001).

## 4. Discussion

We hypothesized that a significant proportion of patients receiving TNK/tPA in the setting of acute ischemic stroke at our facility would not require critical care; our findings support this hypothesis. We found that only around one fifth of our study population met the study definition of requiring an ICU level of care. Furthermore, differences in characteristics between groups, namely the NIHSS at presentation and concurrent antithrombotic management, may inform a care pathway by which carefully selected patients can be observed safely after thrombolytic therapy in a non-ICU care unit setting.

Our findings are concordant with other studies showing patients can often be observed under non-ICU levels of care for part of the 24 h monitoring period following TNK/tPA administration in the setting of acute ischemic stroke. A study conducted by Cencer et al. evaluated the safety and efficacy of a standard 24 h ICU monitoring period compared with a shorter 12 h ICU monitoring period. The study specifically analyzed patients with a diagnosis of acute ischemic stroke who received tPA and who had an admission NIHSS of 0–5 [5]. Baseline demographics including age, gender, and admission NIHSS were not significantly different between the 24 h versus the 12 h ICU monitoring period groups. They found that patients treated within the 12 h ICU protocol had a significantly shorter hospital length of stay. Most importantly, they found that the 12 h ICU monitoring period, compared to the 24 h ICU monitoring period, was not associated with any increase in adverse outcomes. These results suggest that reducing ICU length of stay among a specific subset of acute ischemic stroke patients is a safe practice.

We found that patients with lower initial and post-thrombolytics NIHSS were less likely to have intensive care needs in the 24 h monitoring period after TNK/tPA administration. This finding has been replicated in other similar studies. A prospective study conducted by Fukuda et al. [6] utilized a “fast track protocol” to manage a select subset of acute ischemic stroke patients following the administration of TNK/tPA. This “fast track protocol” involved 12 h of ICU monitoring with neurological checks of decreasing frequency conducted during those 12 h. The protocol was utilized in a subset of patients with a presenting NIHSS < 10, who met the following criteria: no requirement of IV antihypertensive medications to maintain blood pressure below 180/105, no large or medium vessel occlusion, no indication for thrombectomy or non-invasive angiography, no flow limiting extra- or intracranial stenosis of the carotid artery, nor hemodynamic or respiratory instability. These inclusion criteria for the enrollment of patients into the “fast track protocol” were similar to the stated definition of requiring an ICU level of care that was used in our study. Additionally, Fukuda et al. found that among patients treated under the “fast track protocol” there were no cases of sICH and none of these patients needed to be transferred back to the ICU for the development of critical care needs. The results of this study suggest that a certain subset of patients, particularly those with a low initial NIHSS and a lack of initial critical care needs, can be treated safely in a non-ICU setting for a portion of the mandatory observation period following TNK/tPA administration.

The OPTIMIST trial, conducted by Faigle et al., [7] assessed the safety of a low-intensity monitoring protocol in the observation of acute ischemic stroke patients post-TNK/tPA administration. Like the other current literature, this low-intensity monitoring protocol was used to observe patients who had an NIHSS < 10 at the time of presentation and who had no critical care needs by the time the IV thrombolysis infusion ended. This low-intensity monitoring protocol utilized current standard of care protocols for the first hour and then provided care to these patients in a designated stroke/telemetry unit with a nurse-to-patient ratio of 1:3 but with no critical or intermediate care capabilities. Faigle et al. found that none of the patients treated under this protocol required transfer to the ICU or a critical care intervention in the first 24 h following IV thrombolysis. Similarly to what was suggested by our data, the results of this study suggest that a subset of these patients can be monitored safely in a non-ICU setting without critical care abilities.

The findings of other previous studies suggest that blood pressure monitoring, specifically the presence of elevated blood pressure, can be helpful in predicting the need for critical care. One study specifically found that, if blood pressure was not elevated in the first six hours after TNK/tPA administration, then elevation in blood pressure over the next 18 h of the monitoring period was unlikely [8]. The results of this study suggested that not all patients require hourly blood pressure monitoring after TNK/tPA administration for the entire 24 h monitoring period. These results could serve to inform future directions for our study, especially because our patient population tended to be older and consisted of a fair number of patients with pre-existing hypertension.

Our study results suggested that patients on previous antiplatelet therapy were more likely to require an ICU level of care following TNK/tPA administration. Similarly, other studies have also found that patients on antiplatelet therapy with acute ischemic stroke treated with TNK/tPA had a greater risk of developing subsequent sICH compared to those not on antiplatelet therapy [9]. These patients likely represent a subset of patients who may benefit from being treated under current protocols that include the entire 24 h monitoring period in an ICU setting. This same study by Xian et al. found that, although these patients on antiplatelet therapy at the time of arrival were at increased risk of developing subsequent sICH, they were found to have better functional outcomes, measured as independent ambulation and discharge Modified Rankin Score (mRS), than those not on antiplatelet therapy. These results show the importance and benefit of ICU-level monitoring of these patients during an early high-risk period as it may lead to better outcomes.

Interestingly, Xian et al. [9] also investigated whether these risks and benefits of TNK/tPA administration in those on antiplatelet therapy change depending on which antiplatelet agents the patient is on. They found that the highest odds of sICH were among those receiving aspirin alone and among those receiving dual antiplatelet therapy of aspirin and clopidogrel but only reported how other outcomes such as in-hospital mortality, independent ambulation, and mRS score differed between those who were and were not receiving antiplatelet therapy. They did not separate these outcomes by which antiplatelet agents the patient was on. These results, as well as future studies, aimed at further investigating the relationship between the type of antiplatelet agent used and various outcome variables which could be invaluable in better discerning which patients are at increased risk of sICH and other complications of TNK/tPA administration. This would further characterize who would benefit from a full monitoring period in an ICU setting.

It is important to recognize the impact that these findings have in terms of the delivery of safe and effective care while also optimizing healthcare resource utilization. Average daily ICU costs in the United States are commonly estimated at several thousand dollars per day, with detailed analyses reporting mean ICU daily costs around $7700 per day [10]. Routine ICU admission for all post-thrombolysis patients, regardless of their individual risk of complications, would unnecessarily increase healthcare expenditures and place further strain on already limited critical care resources. Our study findings suggest that selective use of non-ICU settings such as progressive care or step-down units has the potential to be both clinically sound and utilize resources more efficiently. Reducing unnecessary ICU utilization could help preserve ICU capacity for higher-acuity patients and decrease overall costs. While this study was not designed as a formal economic analysis, these findings support further investigation into risk-stratified post-thrombolysis care pathways aimed at delivering high-value and cost-effective care. There is an ongoing discussion with our institutional leadership on how best to implement such a protocol given our findings.

Our study has limitations. This study was retrospective in nature, and therefore we were unable to randomize patients to different treatment protocols. We were also dependent upon documentation in the electronic medical record of adverse events and their time course in terms of the accuracy of our data. Additionally, we performed this study at a single thrombectomy-capable stroke center serving patients in rural and suburban Pennsylvania. Our hospital also serves a very large geriatric population, and this population was well-represented in the present study, so our findings may not be generalizable to all centers. Because of the single-center design of our study, the older patient population included, and the lack of functional outcome measures examined, clinical implications may not be generalizable. Our interpretations reflect the population and constraints of our study.

We separated and analyzed our data based on those who were on antiplatelet therapy prior to arrival and those who were not, but we did not assess which antiplatelet medications each patient was on. This is something we would aim to address in future studies, as perhaps there exist differences in outcomes based on the antiplatelet agents used. Additionally, due to the retrospective nature of the study and the challenging nature of completing follow-up appointments during the COVID-19 pandemic, we were unable to assess mRS at discharge and 90-day mRS. In future prospective studies, we would collect these data points, as they are the standard metrics in assessing the functional outcomes of patients following their acute ischemic stroke. Our inability to examine the long-term results of thrombolytic therapy for AIS patients and the efficacy of different treatment modalities is also a limitation that could prove to be an advantageous future direction for our research. Another future direction to consider is potential biomarkers that can serve to personalize interventions and decrease procedure complications for acute ischemic stroke patients.

Mechanical thrombectomy represents an important potential confounder in the interpretation of post-thrombolysis ICU utilization. Patients who undergo thrombectomy often have more severe strokes, larger vessel occlusions, and higher baseline NIHSS scores, all of which would likely independently increase the likelihood of ICU admission and need for critical care interventions [11]. In our study, inclusion of patients who underwent mechanical thrombectomy may, therefore, have increased the observed rate of need for ICU utilization and could overestimate the proportion of post-thrombolysis patients requiring an ICU level of care. Patients treated with thrombectomy likely represent a clinically distinct subgroup that could potentially have different monitoring needs. Future studies may benefit from stratifying patients by thrombectomy status or performing sensitivity analyses excluding patients who underwent mechanical thrombectomy to more precisely define ICU needs attributable specifically to thrombolytic therapy. Such an approach would more specifically define which post-thrombolysis patients can be safely managed outside of an ICU setting.

The results of the present study suggest that there is a subset of acute ischemic stroke patients that can be safely monitored in a non-ICU setting following TNK/tPA administration. We found that patients on previous antithrombotic therapy and those with a higher initial NIHSS were more likely to require an ICU level of care. Based on these results, further studies are warranted to better ascertain the specific characteristics of those patients who could be monitored safely in a non-ICU setting. Prospective studies would aid in the identification of these characteristics. We are uniquely positioned to contribute to this body of literature as our facility is not an academic center, but rather a community-based stroke program with a team composed of attending neurologists, ICU physicians, and advanced practice providers. Future studies could aim to develop and refine a protocol that could be used to aid in the decision of where the patient can be monitored safely following TNK/tPA administration in our community hospital.

## Figures and Tables

**Table 1:** Characteristics and outcomes of patients who required intensive care unit level of care compared to those who did not.

	Did Not Require ICU Level of Care (n = 208)	Required ICU Level of Care (n = 54)	*p*-Value
Age ^a^	71.5 (63–82)	74.5 (67–83)	0.4306
Hispanic/Latino ^b^	16 (7.69%)	7 (12.96%)	0.223
White ^b^	194 (93.27%)	53 (98.15%)	0.553
Male Gender ^b^	76 (36.53%)	26 (48.15%)	0.721
In-Hospital Mortality ^b^	5 (2.40%)	13 (24.07%)	<0.001
Stroke Mechanism ^b,^*			
Small Vessel	33 (97.05%)	1 (2.95%)	0.006
Cardioembolic	54 (72.97%)	20 (27.03%)	0.107
Larger Artery	86 (71.07%)	35 (28.93%)	0.002
Other Identified Source	2 (100.00%)	0 (0.00%)	0.469
Unclear Etiology	86 (85.15%)	15 (14.85%)	0.068
Antithrombotic Use ^b^	64 (30.77%)	31 (57.41%)	<0.001
Stroke on Presentation ^b^	204 (98.08%)	47 (87.04%)	<0.001
Mechanical Thrombectomy ^b^	16 (7.69%)	23 (42.59%)	<0.001
Time from Symptom Onset to TNK/tPA Administration (minutes) ^a^	105 (80–139)	106 (77–155)	0.1795
Systolic Blood Pressure at Time of Administration ^a^	163 (145.5–174)	156 (140–166)	0.1717
Initial NIHSS ^a^	6 (3–11)	13 (7–18)	<0.001
NIHSS Following Administration ^a^	3 (1–7)	13.5 (6–20)	<0.001
Patient Outcomes			
Discharge Destination ^b^			<0.001
Home	123 (59.13%)	8 (14.8%)	
Skilled Nursing Facility	26 (12.50%)	8 (14.8%)	
Rehabilitation Center	48 (23.08%)	13 (24.07%)	
Hospice	3 (1.44%)	9 (16.67%)	
30-Day Readmission ^b^	14 (6.73%)	2 (3.70%)	0.684
Hospital Length of Stay ^a^	3 (2–5)	6.5 (4–12)	<0.001
ICU Length of Stay ^a^	1 (1–2)	3 (1–7)	<0.001

(ICU, intensive care unit; TNK, Tenecteplase; tPA, tissue plasminogen activator; NIHSS, NIH stroke scale). ^a^ median (IQR), ^b^ n (%). * Some patients were categorized as having more than one stroke mechanism, so the mechanisms combined is greater than the total N for the groups.

**Table 2:** Multivariable logistic regression showing risk of requiring intensive care. Unit level of care.

	Odds Ratio	Confidence Interval	*p*-Value
Age	0.976	0.949–1.003	0.080
Race	0.365	0.052–2.559	0.310
Sex	0.663	0.330–1.335	0.250
Stroke Type			
Small Vessel	0.191	0.022–1.676	0.135
Cardioembolic	1.125	0.501–2.525	0.775
Larger Artery	1.069	0.389–2.938	0.896
Unclear Etiology	0.889	0.303–2.607	0.830
Other	0.943	0.295–2.711	0.972
Antithrombotic Use	3.344	1.544–7.242	0.002
Initial NIHSS	1.116	1.062–1.173	<0.001

NIHSS, NIH stroke scale.

## Data Availability

No new data were created or analyzed in this study.

## Abbreviations

The following abbreviations are used in this manuscript:

AIS	Acute Ischemic Stroke
ICU	Intensive Care Unit
TNK	Tenecteplase
tPA	Tissue Plasminogen Activator
AOR	Adjusted Odds Ratio
NIH	National Institute of Health
sICH	Symptomatic Intracerebral Hemorrhage
IV	Intravenous
IRB	Institutional Review Board
NIHSS	National Institute of Health Stroke Scale
